# High sensitivity and specificity rates of cobas® HPV test as a primary screening test for cervical intraepithelial lesions in a real-world setting

**DOI:** 10.1371/journal.pone.0279728

**Published:** 2023-02-06

**Authors:** Laura Alicia Fleider, María de los Ángeles Tinnirello, Facundo Gómez Cherey, María Gabriela García, Lucía Helena Cardinal, Florencia García Kamermann, Silvio Alejandro Tatti

**Affiliations:** 1 OBGYN Department, Genital Tract Unit, Hospital de Clínicas “José de San Martín”, Buenos Aires University, Buenos Aires, Argentina; 2 Molecular Infectious Disease Department, ManLab Laboratories, Buenos Aires, Argentina; 3 Gynecological Pathology Division, Pathology Department, Hospital de Clínicas “José de San Martín”, Buenos Aires University, Buenos Aires, Argentina; 4 Chief of OBGYN Department, Hospital de Clínicas “José de San Martín”, Buenos Aires University, Buenos Aires, Argentina; Teikyo University, School of Medicine, JAPAN

## Abstract

Cervical carcinoma (CC) is the fourth most common malignancy among women. Screening with Papanicolau smear is linked to a reduction in CC incidence rates when screening programs have been developed. However, this technique has several limitations, including moderate sensitivity rates for detection of cervical preneoplastic HPV-related lesions. In this real-world study, we proposed to evaluate the sensitivity and specificity rates of cobas® test, which amplifies target DNA fragments by polymerase chain reaction and hybridization of nucleic acids for the detection of 14 HR-HPV types in a single analysis) used as primary screening test for CC and preneoplastic lesions in women aged 25–65 years in a large University Hospital in Buenos Aires. A total of 1044 patients were included in the sample (median age: 46 years); sensitivity and specificity rates for the HR-HPV test used as primary screening test were 98.66% (95% confidence interval [95CI]: 97.67–99.3%) and 87.15% (95CI: 84.93–89.15%), respectively. The positive predictive value was 88.47% (95CI: 86.54%-90.42%) and the negative predictive value was 98.48% (95CI: 97.75%-99.23%). The cobas® HR-HPV testing was highly sensitive and specific for the detection of CC and preneoplastic lesions in real practice.

## Introduction

According to current global data, cervical carcinoma (CC) is the fourth most common malignancy among women. In 2018, an estimated 570,000 women were diagnosed with CC worldwide, and about 311,000 women died from this disease [1]. It is worth noting that primary and secondary prevention approaches will prevent more CC cases. Screening with Papanicolaou (PAP) smears is linked to a striking reduction in CC incidence rates when screening programs have been developed.

PAP cervical cytology is a recognized tool to evaluate exfoliated cervical cells in order to detect potential morphological changes indicative of preneoplastic and neoplastic alterations, leading to lower rates of CC through early detection and treatment of precursor lesions. Nevertheless, the PAP smear also has several limitations, including moderate sensitivity rates for the detection of cervical intraepithelial neoplasia (CIN) grade 2 or 3 (CIN2/3) and adenocarcinoma. Therefore, cervical cytology needs to be systematically repeated. Poor sensitivity for the detection of adenocarcinoma preneoplastic lesions has been associated with the scarcity of exfoliated cells obtained from the endocervical canal [2]. Other limitations of PAP screening are subjectivity bias due to low reproducibility [3], and the usually high working load in pathology laboratories contrasting with a reduced staff.

Under the paradigm that human papillomavirus (HPV) infection is necessary for preneoplastic and CC development [4], tests to detect infected patients have allowed to develop new and highly cost-effective strategies intended for the prevention of this disease. Laboratory tests for the detection of high-risk (HR) HPV are more sensitive [5, 6] and reliable [7, 8] than conventional cytology for the detection of preneoplastic lesions and CC. A negative HR-HPV test has a high negative predictive value (NPV), which may lead to longer intervals between screening visits, and reduced incidence and mortality due to CC [9].

Screening with the HR-HPV test versus conventional cytology alone were compared in at least 4 randomized controlled trials recruiting over 250,000 women: the New Technologies for Cervical Cancer (NTCC) Phase II trials [10, 11], the HPV for Cervical Cancer Screening (HPV FOCAL) trial [12], the FINNISH trial [13], and the COMPASS trial [14]. Overall, these four trials concluded that HR-HPV tests alone led to an increase in the CIN3 detection rate when compared with cytology alone in a first detection round. Colposcopy rates were higher [11] or similar [12, 13] among patients in the HR-HPV arm when compared to women who were diagnosed by cytology alone. False positive rates for CIN ≥ 2 (CIN2+) were higher [10] or similar [13] for HR-HPV testing than for conventional cytology.

Other specific molecular biomarkers are associated with high sensitivity and specificity rates for the detection of cervical squamous intraepithelial lesions (SIL) [15]. Progression from HPV infection to CC is characterized by an important change in viral oncogenes expression impacting on cell cycle checkpoints [16].

In this study, we proposed to evaluate the sensitivity and specificity rates of HR-HPV test as a primary screening test for CC and preneoplastic lesions in women aged 25–65 years in a large University Hospital in Buenos Aires.

## Materials and methods

### Design

This prospective cohort study was performed in the *Hospital de Clínicas “José de San Martín”*, a large University Hospital in Buenos Aires. Inclusion criteria were: (1) stated sexual intercourse; (2) aged 25 to 65 years old; (3) able to sign an informed consent. Exclusion criteria were: (1) pregnancy; (2) hysterectomy for any reason; (3) normal menses or abnormal uterine bleeding on the day of cervical sample collection; (4) lower genital tract, vaginal or uterine malformations; (5) less than 48 hours of sexual abstinence; (6) actual diagnosis of CC.

### Objectives

Our main objective was to calculate the sensitivity and specificity rates of the HR-HPV test as a primary screening test for CC and preneoplastic lesions. Secondary objectives included evaluating the prevalence of HPV-related lesions in the targeted population, estimating the rate of necessary colposcopies under this screening/triage program, and evaluating the mean age of patients with CIN3 in this population.

### Patients’ journey

After signing the informed consent, patients who agreed to participate were evaluated in an initial visit to obtain a complete general and gynecological medical record. Two vaginal samples were obtained for cytology (Ayre spatula and cytobrush for exo- and endocervical study). Conventional cytology samples were processed in the Department of Pathology by Papanicolaou staining, and reported according to the current Bethesda classification. A different sample obtained with a sterile cytobrush was collected for DNA HR-HPV cobas® PCR Cell Collection Media test. This tool amplifies target DNA fragments by polymerase chain reaction and hybridization of nucleic acids for the detection of 14 HR-HPV types in a single analysis. The test specifically identifies HPV16 and HPV18 genotypes and simultaneously detects other clinically relevant HR types (31, 33, 35, 39, 45, 51, 52, 56, 58, 59, 66, and 68). Patients with positive HPV16/18 cobas® test, atypical glandular cells (AGC), high-grade squamous intraepithelial lesions (HSIL) or ASC-H (atypical squamous cells—cannot exclude HSIL) cytology, or a potentially invasive lesion were referred for colposcopy. Colposcopies were performed with 16x magnification following the application of a solution of 3–5% acetic acid for 3 minutes. Colposcopic images were digitized for follow-up. A vaginal, vulvar, perineal and perianal visual inspection was also performed. All collected images were reported according to the International Federation of Cervical Pathology and Colposcopy Nomenclature (IFCPC, 2011 version) [17]. Patients with grade 1 or higher colposcopy images were biopsied with Tischler biopsy forceps, using previous cervical antisepsis. Hemostasis was performed with Monsel’s solution (ferric subsulfate).

In patients with type 3 transformation zone or an endocervical canal component, an endocervical sample was obtained with cytobrush or Kevorkian curette. Histological samples were reported according to the Lower Anogenital Squamous Terminology (LAST) [18]. All samples were evaluated by expert gynecologic pathologists. Follow-up visits were scheduled on the basis of the obtained results.

### Ethics

This study was approved by the Teaching and Research Department and the Ethics Committee of the *Hospital de Clínicas “José de San Martín”*. Patients could withdraw at any time and for any reason. If lost to follow-up, the assigned study staff tried to contact the subject to establish and document the reason for withdrawal.

### Statistical analysis

The estimated sample size of the whole cohort was 1000 patients, considering an alpha error of 0.05%, a beta error of 0.20%, and 16% of HPV positive tests in 8100 women annually treated in our hospital. All retrieved data were anonymized and tabulated in a Microsoft Excel® spreadsheet. Continuous variables were analyzed by central and dispersion statistics, while categorical variables were described according to their frequency. Sensitivity and specificity were calculated according to conventional formulas. A two-tailed p-value ≤ 0.05 was considered significant for comparisons. A statistical analysis was performed using Epi Data® version 7.2.2.6 (Centers for Disease Control and Prevention, U.S. Department of Health & Human Services).

## Results

**Fig 1:**
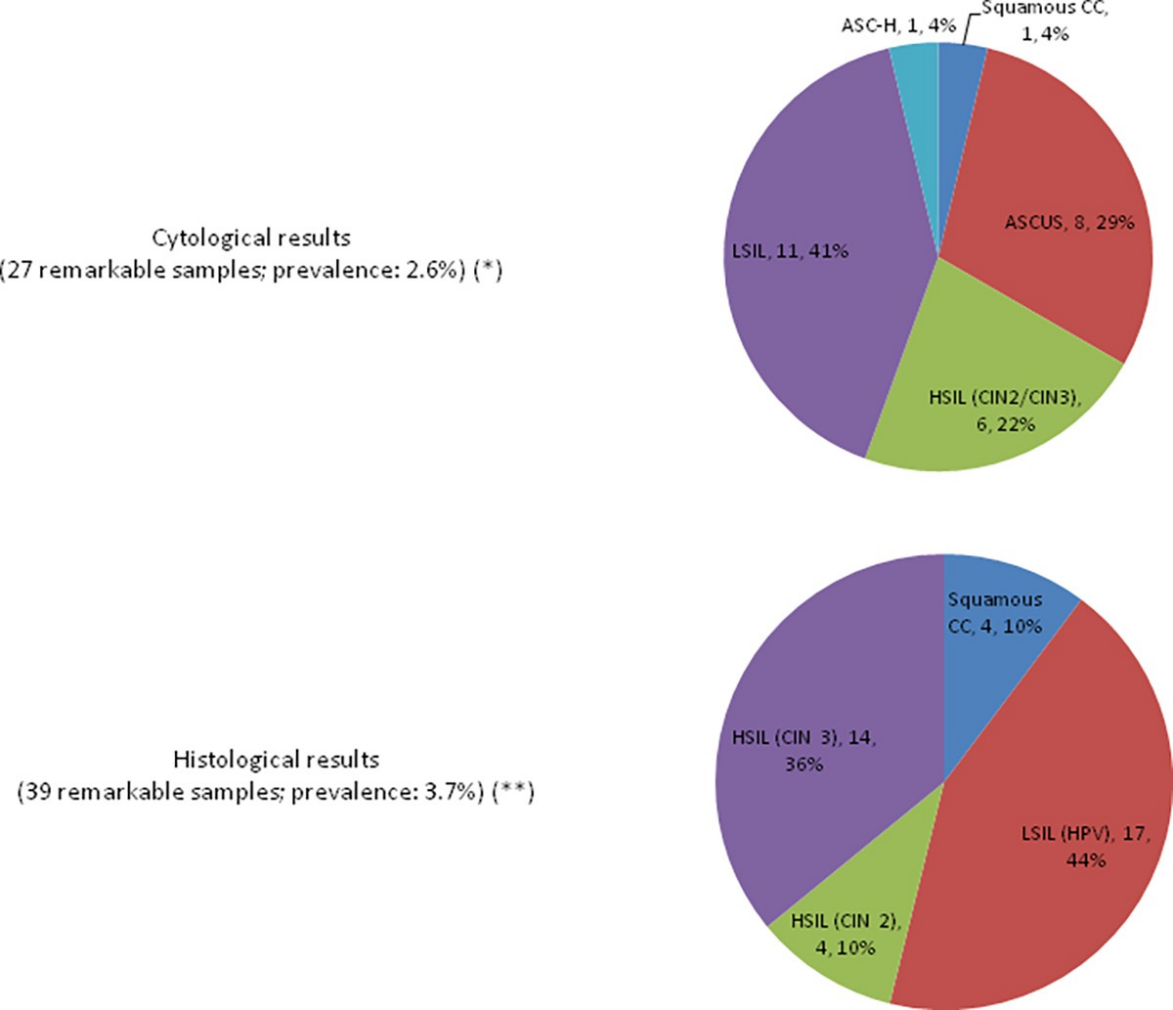
Squamous intraepithelial lesions. (*) Negative, inflammatory and inadequate results were excluded. (**) Negative, inflammatory (including chronic cervicitis) and inadequate results were excluded.

A total of 1143 women were initially evaluated for participation; after considering the inclusion and exclusion criteria, 1044 patients were included in the sample (median age: 46 years, range: 25–65). Cervical cytology and cobas® tests results are summarized in Tables 1 and 2, respectively. Distribution of squamous lesions among studied patients is shown in Fig 1. One hundred and fifty patients were tested positive for HR-HPV by cobas® test, accounting for 14.37% of the whole cohort. Thirty five of these women were tested positive for HPV16 and/or HPV18 alone or in coinfection with other HR-HPV (23.33% of this subgroup).

**Table 1:** PAP results.

Result	n/N (percent)	Cumulative percent	95% confidence interval
Negative	1011/1044 (96.93%)	96.93%	95.88–97.98%
ASC-US	7/1044 (0.67%)	97.60%	0.33–1.01%
ASC-H	1/1044 (0.05%)	97.65%	0.00–0.10%
LSIL	10/1044 (0.96%)	98.61%	0.52–1.75%
HSIL (CIN2/CIN3)	6/1044 (0.57%)	99.18%	0.23–0.91%
Squamous carcinoma	1/1044 (0.05%)	99.23%	0.00–0.10%
Inadequate samples	8/1044 (0.77%)	100%	0.43–1.11%
TOTAL	1044/1044 (100%)	100%	

**Table 2:** Cobas® test results.

Result	n/N (percent)	Cumulative percent	95% confidence interval
Non detectable	894/1044 (85.63%)	85.63%	83.50–87.76%
HPV-16	17/1044 (1.63%)	87.26%	0.86–2.40%
HPV-18	8/1044 (0.77%)	88.03%	0.17–1.37%
Coinfection (*)	10/1044 (0.95%)	88.98%	0.35–1.45%
Other HR-HPV (*)	115/1044 (11.02%)	100%	9.12–12.92%
TOTAL	1044/1044 (100%)	100%	

(*) HPV-16 and/or HPV-18 + other HR-HPV

HR-HPV was detected by the cobas® test in 130 (12.84%) out of 1012 female participants with negative cytology, including 27 cases of HPV16 and/or HPV18 (20.8% of this subgroup). Distribution is fully described in Tables 3–5.

**Table 3:** Comparison between cytological diagnosis and cobas® test results.

		Cytological results							
		Negative	ASCUS	ASC-H	AGC	LSIL	HSIL	Squamous CC	Inadequate /Inflammatory
cobas® test results	Non detectable (n = 894)	882 (98.7%)	2 (0.2%)	0 (0%)	0 (0%)	1 (0.1%)	1 (0.1%)	1 (0.1%)	7 (0.8%)
	HPV-16 (n = 17)	12 (70.6%)	2 (11.8%)	0 (0%)	0 (0%)	2 (11.8%)	0	0 (0%)	1 (5.8%)
	HPV-18 (n = 8)	7 (87.5%)	0 (0%)	0 (0%)	0 (0%)	0	1 (12.5%)	0 (0%)	0 (0%)
	Other HR-HPV (n = 115)	102 (88.7%)	3 (2.6%)	0 (0%)	0 (0%)	6 (5.2%)	4 (3.5%)	0 (0%)	0 (0%)
	Coinfection (n = 10)	9 (90%)	0 (0%)	0 (0%)	0 (0%)	1 (10%)	0 (0%)	0 (0%)	0 (0%)

(*) HPV-16 and/or HPV-18 + other HR-HPV

**Table 4:** Comparison between histological diagnosis and cytological results.

		Cytological results							
		Negative	ASCUS	ASC-H	AGC	LSIL	HSIL	Squamous CC	Inadequate
Histological results	Negative (n = 117)	109 (93.2%)	1 (0.8%)	1 (0.8%)	0 (0%)	5 (4.4%)	1 (0.8%)	0 (0%)	0 (0%)
	L-SIL (n = 17)	5 (29.4%)	5 (29.4%)	0 (0%)	0 (0%)	6 (35.3%)	1 (5.9%)	0 (0%)	0 (0%)
	H-SIL (n = 18)	13 (72.2%)	2 (11.1%)	0 (0%)	0 (0%)	0 (0%)	3 (16.7%)	0 (0%)	0 (0%)
	Squamous CC (n = 4)	1 (25%)	0 (0%)	0 (0%)	0 (0%)	0 (0%)	1 (25%)	1 (25%)	1 (25%)

“Negative” category includes all non-squamous lesions.

**Table 5:** Comparison between histological diagnosis and cobas® test results.

		cobas® test results				
Histological results		Non detectable	HPV-16	HPV-18	Other HR-HPV	Coinfection (*)
	Negative (*) (n = 37)	23 (62.2%)	1 (2.7%)	2 (5.4%)	10 (27%)	1 (2.7%)
	L-SIL (n = 17)	2 (11.8%)	4 (23.5%)	1 (5.9%)	6 (35.3%)	4 (23.5%)
	H-SIL (n = 18)	0 (0%)	4 (22.2%)	1 (5.6%)	9 (50%)	4 (22.2%)
	Squamous CC (n = 2)	1 (50%)	1 (50%)	0 (0%)	0 (0%)	0 (0%)

(*) HPV-16 and/or HPV-18 + other HR-HPV

The sensitivity and specificity rates for the HR-HPV test as a primary screening test were 98.66% (95% confidence interval [95CI]: 97.67–99.3%) and 87.15% (95CI: 84.93–89.15%), respectively.

The unadjusted prevalence rate of HPV-related lesions -including the results from exocervical and endocervical PAP staining and biopsies- was 3.6% (95CI: 2.59–4.96%). All these patients tested positive for HR-HPV using the cobas® test. The positive predictive value was 88.47% (95CI: 86.54%-90.42%), and the negative predictive value was 98.48% (95CI: 97.75%-99.23%).

One hundred and sixteen colposcopies were performed (11.11% of all patients). The cobas® tests were performed according to the described patient journey in 50/116 (43.1%) patients; 46/50 subjects tested positive for at least one HR-HPV type (92%).

Defining the median age in the subgroup of patients with CIN3 was another secondary objective of our research. Ten patients were diagnosed with CIN3 (0.9%), all of them with a positive cobas® test for HR-HPV. Median age among these women was 32 years (range: 26 to 64), significantly lower when compared to the whole cohort (p < 0.01, Mann-Whitney U test).

## Discussion

In this large cohort of women assisted in a University Hospital in Buenos Aires, the cobas® test for HR-HPV was highly sensitive and specific for the detection of preneoplastic lesions and CC. These rates are consistent with the current literature [19, 20]. The proportion of positive tests for HR-HPV was 14.37%; overall, HPV prevalence in the Latin American and Caribbean female population has been reported as twice higher than the average worldwide rate [21]. Follow-up studies have reported that a continuous presence of HR-HPV is necessary for the development, maintenance and progression of CIN. Nevertheless, only a small proportion of women with cervical HPV infection develop CC, suggesting that certain cofactors are necessary for progression [22]. As the previous regional literature and our research show, HPV testing has a greater sensitivity to detect CIN2+ and CC than the PAP smear [23, 24]. HPV typing also improves rates of both false-negative and false-positive cytology results [22]. The unadjusted prevalence of HPV-related lesions in our population was 3.6%, which is lower than the rates reported in other series in the developing world [25] and Latin America [26]. These differences deserve future research.

Of note, all participants with abnormal colposcopy reports tested positive using the cobas® test. Many low-grade lesions will regress spontaneously, and excisional treatment may be associated with adverse events [27].

Even though the reduced sample of CIN3 patients precludes a rigorous analysis, this subgroup of women was significantly younger than the whole cohort. According to previous Argentinean data, HPV infection rates peak below the age of 25, and then drop and plateau around 30–35 years [28]. This pattern resembles the reports of HPV infection in industrialized countries. Persistence of oncogenic HPV has been proposed as more sensitive and specific than cytology for early detection of CIN3 and invasive CC [29].

## Conclusion

In this large sample, cobas® HR-HPV testing was highly sensitive and specific for the detection of CC and preneoplastic lesions. All women with abnormal colposcopies tested positive using the cobas® method. Median age of CIN3 patients was 32 years; It is emphasized that these women of childbearing age require excisional therapy.

## Supporting information

S1 Checklist STROBE (Strengthening The Reporting of OBservational Studies in Epidemiology) checklist. (PDF)
